# Sol–gel encapsulation of Au nanoparticles in hybrid silica improves gold oxidation catalysis

**DOI:** 10.1186/s13065-016-0208-6

**Published:** 2016-10-13

**Authors:** Rosaria Ciriminna, Valerica Pandarus, Riccardo Delisi, Antonino Scurria, Maria Pia Casaletto, Francesco Giordano, François Béland, Mario Pagliaro

**Affiliations:** 1 Istituto per lo Studio dei Materiali Nanostrutturati, CNR, via U. La Malfa 153, 90146 Palermo, PA Italy; 2 SiliCycle Inc., 2500, Parc Technologique Blvd, Quebec City, QC G1P 4S6 Canada

**Keywords:** Gold, Nanoparticle, Alcohol oxidation, Hydrogen peroxide, Sol–gel

## Abstract

**Background:**

The introduction of an heterogeneously catalyzed gold-based alcohol oxidation process of broad applicability using a clean primary oxidant would be highly desirable. Gold is non toxic and carbonyl and carboxyl compounds are widely used to produce medicines, plastics, colorants, paints, detergents, fragrances, flavors, and other valued functional products.

**Results:**

The sol–gel entrapment of gold nanoparticles in hybrid silica improves gold-based oxidation catalysis applied to the selective oxidation of alcohols with aqueous hydrogen peroxide as eco-friendly primary oxidant. Pronounced physical and chemical stabilization of the sol–gel entrapped Au nanoparticles is reflected in catalyst recyclability.

**Conclusions:**

Potential implications of these findings are significant, especially considering that the highly stable, mesoporous glassy catalyst is ideally suited for application in microreactors for carrying out the reaction under flow.

**Graphical abstract:**
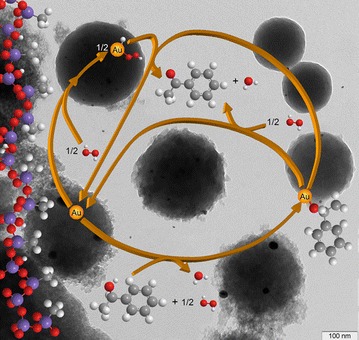
Selective oxidation of 2-phenylethanol over SiliaCat Au nanoparticle. Hydrogen peroxide is the clean oxidant.

Following three decades of intense fundamental research [1], heterogeneous gold catalysis is now applied by the chemical industry to large-scale manufacturing of important monomers, with several Au-based redox syntheses likely to find commercial application in the next few years [2]. Alcohols, in their turn, are starting materials for the synthesis of a variety of aldehydes, ketones and carboxylic acids which are then used to produce medicines, plastics, colorants, paints, detergents, fragrances, flavors, and other valued functional molecules [3]. To accomplish this fundamental reaction, for several decades prior to the recent introduction of heterogeneously catalyzed processes [4], industry has relied on alcohol stoichiometric oxidation with inorganic and organic hazardous oxidants [5].

The ideal oxidant, though, is air or aqueous hydrogen peroxide (added slowly to avoid quick metal-catalyzed decomposition) [6], preferably under solvent-free conditions; whereas the ideal catalyst is non toxic and extensively recyclable. In this respect, the use of gold in place of platinum group metals, especially Pt and Pd, would be particularly advantageous as many aldehydes and ketones products of the oxidative conversion are active pharmaceutical ingredients (APIs) and the latter metals are classified as ‘Metals of significant safety concern’ with allowed residues in APIs <10 ppm if orally administered, and <1 ppm if parenterally administered [7]. Gold, on the other hand, is of limited toxicity.

A major progress in the field occurred in 2009 when Cao and co-workers in China reported that commercial catalyst AURO*lite* comprised of 1 % Au/TiO_2_ is an efficient and reusable catalyst for the base-free and solvent-free oxidation of alcohols using aqueous H_2_O_2_ as primary oxidant at 90 °C [8]. In Cao’s oxidation system (H_2_O_2_–Au/TiO_2_), secondary alcohols are converted into ketones, whereas primary alcohols are oxidised to carboxylic acids.

Along with Ilharco and Fidalgo, we have recently reported that a catalyst comprised of Au nanoparticles (NPs) sol–gel entrapped in the aggregated mesoporosity of 5 %-methyl modified silica (trademarked Silia*Cat* Au) selectively catalyzes the oxidation of 1-phenylethanol to acetophenone [9] under the same base- and solvent-free conditions developed by Cao’s team in 2009. Now we report that this hybrid catalyst can be successfully applied to oxidise various secondary or primary alcohols, with striking selectivity differences when compared to Au/TiO_2_, while retaining its activity in several consecutive runs.

Table 1 compares the reactivity of the catalyst under different conditions for different substrates giving the yields after 3 h of reaction. Entry 1 shows that cinnamyl alcohol is selectively oxidised to cinnamaldehyde only. We repeated then the oxidation of 1-phenylethanol, using 1.5 equiv of H_2_O_2_. This time, the yield is reduced from 94.2 % (entry 2, when using 3.2 equiv of H_2_O_2_) to 69.5 % (entry 3). The oxidation of 4-methylbenzyl alcohol, namely of a benzylic alcohol with an electron donating group, affords 31 % of aldehyde though 6 % of the corresponding carboxylic acid is also obtained (entry 4). Remarkably, the oxidation of another substrate bearing an electron donating group such as the methoxy substituent in *para* position in the benzene ring (entry 5) affords 80 % aldehyde, and the same amount of acid (6 %).

**Table 1:** Oxidation of alcohols with H_2_O_2_ over Silia*Cat* Au

Entry	Substrate	Au (mol%)	H_2_O_2_ (eq.)	Yield	Time (h)
1	Cinnamyl alcohol	0.5	3.2	53.6 % (aldehyde only)	3
2	1-phenylethanol	0.5	3.2	94.2 % (ketone only)	3
3	1-phenylethanol	0.5	1.5	69.5 % (ketone only)	3
4	4-methylbenzyl alcohol	1.0	3.2	31 % (aldehyde) 6 % (acid)	3
5	4-methoxybenzylalcohol	1.0	3.2	80 % (aldehyde) 6 % (acid)	3
6	1-nonanol	1.0	3.2	13 % (aldehyde) 4 % (acid)	3
7	Cyclohexanol	1.0	3.2	10 % (ketone)	3

*Reaction conditions*: 10 mmol substrate, 10 mL H_2_O, 90 °C, 5 % H_2_O_2_ added dropwise during 30 min; the catalyst amount was determined following ICP-MS analysis of the Au load in the catalyst

Finally, the oxidation of primary aliphatic alcohol 1-nonanol (entry 6) mostly affords the aldehyde (13 %) and a limited amount of acid (4 %); whereas the oxidation of cyclohexanol affords 10 % of cyclohexanone as the only oxidation product (entry 7). Reactions in general proceed with high degree of conversion for most substrates by just prolonging the reaction time, though at lower rate than with Au/TiO_2_ [8]. For example, after overall 12 h of reaction, cyclohexanol is converted in cyclohexanone in 83 % yield.

These findings confirm a peculiar characteristic of the organically modified silica (ORMOSIL) [10] catalyst which is due to the hydrophobized nature of the embedding matrix, namely that in the oxidation of primary alcohols aldehydes are preferably formed, rather than carboxylic acids. This aspect further differentiates the Silia*Cat* Au catalyst from Au/TiO_2_ which, under similar reaction conditions, preferably affords carboxylic acids. Such enhanced hydrophobicity, and the chemical sponge nature of the sol–gel encapsulating matrix (whose large inner meoporosity favours diffusion of incoming H_2_O_2_ molecules), also explains why slightly more than 3 equiv of H_2_O_2_ are required for optimal activity rather than 1.5 such as in the case of Au/TiO_2_, with the catalytic activity of entrapped gold nanoparticles for H_2_O_2_ decomposition being greatly influenced by the ease of adsorption of the molecules over the Au NPs *and* by the electron transfer from Au to H_2_O_2_ [11] both of which are enhanced in the case of Silia*Cat* Au.

Accordingly, Table 2 shows that recycling of the catalyst in the oxidation of 4-methoxybenzyl alcohol progressively affords more acid reaction product, going from 6 to 13.2 % (entries 1 and 3). We ascribe this finding to progressive accumulation of water molecules which partly remain entrapped within the sol–gel cages with ongoing reaction and washing cycles, as the catalyst after each run is washed with water (4 × 15 mL) and then with EtOAc (3 × 20 mL) prior to drying in an oven at 60 °C overnight.

**Table 2:** Oxidation recycles of two alcohol substrates with H_2_O_2_ over Silia*Cat* Au

Entry	Substrate	Au (mol%)	H_2_O_2_ (eq.)	Yield	Recycle
1	4-methoxybenzyl alcohol	1.0	3.2	80 % (aldehyde) 6 % (acid)	1
2	4-methoxybenzyl alcohol	1.0	3.2	68.6 % (aldehyde) 10.4 % (acid)	2
3	4-methoxybenzyl alcohol	1.0	3.2	62.5 % (aldehyde) 13.2 % (acid)	3
4	1-phenylethanol	0.5	1.5	69.5 % (ketone only)	1
5	1-phenylethanol	0.5	1.5	70.3 % (ketone only)	2
6	1-phenylethanol	0.5	1.5	68.4 % (ketone only)	3

*Reaction conditions*: 10 mmol substrate, 10 mL H_2_O, 90 °C, 5 % H_2_O_2_ added dropwise; substrate:H_2_O_2_:Au = 100:150:1. The catalyst amount was determined following ICP-MS analysis of the Au load in the catalyst

Finally, the results of the consecutive oxidation of 1-phenylethanol with the same catalyst washed and reused show that the catalyst is fully recyclable with no loss in activity between subsquent reaction runs (entries 4–6).

The Silia*Cat* Au catalyst is comprised of agglomerates or compacts of spheroidal silica microparticles 100–400 nm in diameter with a large concentration in 7–20 nm Au nanoparticles [9]. The vast inner mesoporosity (broad range of mesopore with average dimension ~20 nm) clearly shown in the TEM microphotograph (Fig. 1) originates the large specific surface area (460 m^2^ g^−1^) and total pore volume (1.49 cm^3^ g^−1^).

**Fig. 1:**
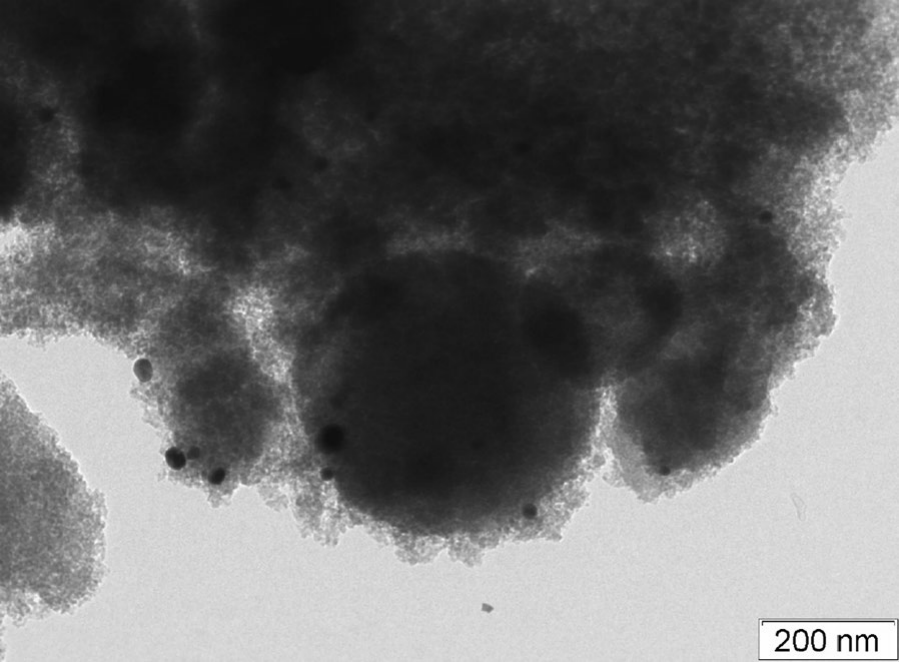
TEM image of SiliaCat Au (5 % methylated)

Contrary to Pd(0) catalyst of the Silia*Cat* series [12], the encapsulation process requires the presence of polyvinyl alcohol (PVA) with practically all valued gold in the HAuCl_4_ precursor ending entrapped in the final xerogel, as proved by the load valued assessed via ICP-MS on the final xerogel. The relatively mild calcination step at 250 °C aims to eliminate the PVA used to prevent agglomeration of the AuNPs during the material’s synthesis. The XRD analysis of Silia*Cat* Au before calcination at 250 °C (Fig. 2) reveals the presence of the reflections of metallic Au (fcc structure). No changes in the Au crystallite size occurred upon calcination, as estimated by the Scherrer equation.

**Fig. 2:**
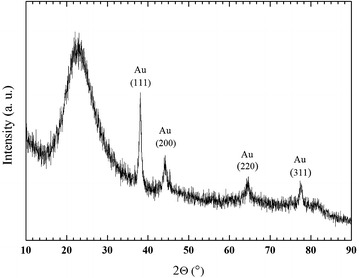
XRD pattern of Silia*Cat* Au catalyst

The XPS pattern in Fig. 3 reveals the surface chemical composition of the ORMOSIL surface. The Si 2p photoelectron peak is located at a binding energy (BE) of 103.8 eV and the O 1 s peak is detected at BE = 532.7 eV [13]. The C 1 s peak at BE = 285.1 eV corresponds to the presence on the surface of carbon atoms in C–C, C–H bonds.

**Fig. 3:**
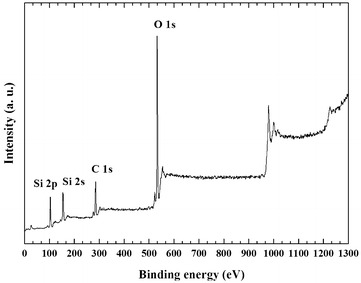
XPS wide spectrum of Silia*Cat* Au catalyst

A very feeble Au 4f photoelectron signal is detected, suggesting that only traces of gold are present on the surface of the samples, in agreement with the nature of the sol–gel encapsulation phenomenon in which the dopant species are entrapped *within* the sol–gel cages [14]. The thermal treatment at 250 °C of the as-prepared sol–gel catalyst is at least partly effective in the removal of the PVA, leading to a direct exposure of the surface of the hybrid silica matrix. Indeed, results of the XPS surface quantitative chemical analysis before and after calcination show an increase of the Si concentration and a corresponding decrease of the C atomic percentage upon calcination, as shown in Table 3.

**Table 3:** XPS surface chemical composition of Silia*Cat* Au before and after calcination. Elemental concentration is expressed as  % atomic percentage

Sample	C 1 s (%)	O 1 s (%)	Si 2p (%)	Au 4f_7/2_	Si/O	Si/C
Silia*Cat*Au	14.6	61.3	24.1	0.1	0.39	1.65
Calcined Silia*Cat*Au	12.0	61.8	26.2	0.1	0.42	2.18

The XPS curve-fitting of the Si 2p spectrum of the Silia*Cat* Au sample inserted in Fig. 4 reveals the presence of second component at BE = 105.1 eV (14.4 % peak area), along with the principal component at BE = 103.8 eV assigned to Si–O bonds (85.6 % peak area). The former component at BE = 105.1 eV is attributed to the presence of surface Si–CH_3_ bonds, which are prevalent on the surface of a fully methylated ORMOSIL matrix, when said component at higher binding energy becomes predominant in the Si 2p curve-fitting, as shown in Fig. 5.

**Fig. 4:**
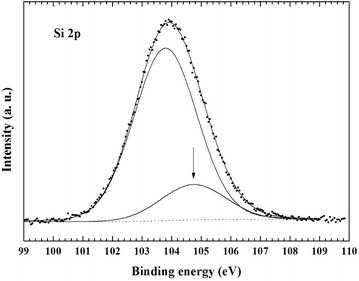
XPS curve-fitting of the Si 2p spectrum of the Silia*Cat* Au sample

**Fig. 5:**
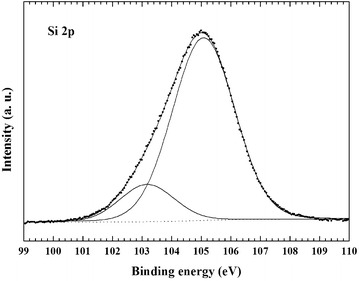
XPS curve-fitting of the Si 2p spectrum of the fully methylated ORMOSIL sample

The removal of the non entangled PVA molecules by calcination yields sol–gel cages with surfaces covered by a population of methyl groups (and weakly interacting –OH groups). These groups are concentrated at the sol–gel cage external surface, where they enhance the hydrophobicity and the electron density at the surface of the entrapped Au nanoparticles. This favors the first mechanistic steps in the gold-mediated oxidation of alcohols with H_2_O_2_, namely the dissociation of H_2_O_2_ and formation of the alcoholate [8].

In conclusion, Silia*Cat* Au successfully mediates the clean oxidation of various alcohols under solvent-free and base-free conditions to the corresponding carbonyl compounds (ketones and aldehydes) using aqueous hydrogen peroxide as primary oxidant at 90 °C. The catalyst has activity comparable to state of the art (AURO*lite*) Au/TiO_2_ catalyst, but the selectivity is reversed, with formation of aldehyde favored also in the case of primary alcohols due to the hydrophobized nature of the encapsulating matrix.

Beyond interesting applications in photonics due to entrapped plasmonic nanoparticles [15], the robust and highly porous nature of the glassy organosilica matrix of the Silia*Cat* catalyst is ideally suited for application to reaction in microreactors under flow [16]. Silia*Cat* Au, with its high activity and particularly pronounced chemical stability, may be ideally suited to provide the fine chemical and pharmaceutical industries with an economically and technically viable solution to oxidise alcohols getting rid of harmful oxidants and toxic catalytic species altogether.

## Experimental section

### Catalyst preparation

The Silia*Cat* Au catalyst was prepared via sol–gel hydrolytic polycondensation of tetraethoxysilane/methyltriethoxysilane (TEOS/MTES) mixtures, in the presence of HAuCl_4_, and PVA as protecting agent. A mixture of TEOS (95 mol %) and MTES (5 mol %) is hydrolyzed by treatment with HCl 0.05 N. A solution of HAuCl_4_ monohydrate (0.20 g) in 3 wt% aqueous PVA is then added with NaBH_4_ (0.05 g), followed by addition of the as-obtained Au nanoparticles to the hydrolized silicon alkoxides mixture. Gelation is promoted through the addition of aqueous NaOH. The purple alcogel thereby obtained is left to dry at room temperature and then powdered. PVA was removed by calcination at 250 °C, with the methyl groups in the organosilica matrix being thermally stable [17]. The catalytic load, obtained with an ICP-MS instrument (Agilent Technologies 7500ce Series Spectrometer) equipped with a collision cell, was 0.1 wt% Au.

### General oxidation procedure

The oxidation of all alcohol substrates was carried out with aqueous H_2_O_2_ (5 wt%) as oxidant over the Silia*Cat* Au catalyst. No organic solvent was employed and air was not excluded from the reaction mixture. In a typical catalytic run, 5 % H_2_O_2_ (18 g) was added dropwise to a suspension of the catalyst (Au 1 mol %) in a mixture of substrate (1 mmol) and deionized water (10 g) contained in a 50 mL two-neck round bottom flask, kept at 90 °C using a thermostatic oil bath. After 3 h the catalyst was filtered and the aqueous reaction mixture extracted with ethyl acetate (EtOAc). The organic phase was then analyzed by GC-FID (Shimadzu-GC 17 equipped with a Supelcowax 10 column) to determine the conversion in carbonyl (or carboxyl) compound. The catalyst was extensively washed (with water and then with acetonitrile), dried at 60 °C overnight and reused as such in a subsequent reaction run.

## Structural analyses

The geometric structure of the samples was investigated by X-ray Diffraction (XRD) by using a Bruker D5000 powder diffractometer, equipped with a Ni-filtered radiation of the Cu anode (Cu K_α_ λ = 1.5418 Å; 40 kV and 35 mA). Diffraction patterns were registered in the angular range 10° < *2θ°* < 90°, using a step size of 0.03° and a time step of 3 s. The assignment of the crystalline phases was based on the ICSD database.

The surface chemical composition of the samples was analyzed by an advanced surface-sensitive technique, such as X-ray photoemission spectroscopy (XPS), by using a VG Microtech ESCA 3000 Multilab spectrometer, equipped with a standard Al K_α_ excitation source (hν = 1486.6 eV) and a nine-channeltrons detection system. The hemispherical analyser operated in the CAE mode, at a constant pass energy of 20 eV Photoemission spectra were collected in an ultrahigh vacuum (UHV) chamber with a base pressure in the range of 10^–8^ Torr during data collection. The binding energy (BE) scale was calibrated by measuring C 1 s peak (BE = 285.1 eV) from the surface contamination and the accuracy of the measure was ±0.1 eV. Data analysis was performed by a nonlinear least square curve-fitting program (VGX900 software) using a properly weighted sum of Lorentzian and Gaussian component curves, after background subtraction according to Shirley and Sherwood [18].

The TEM micrographs were obtained by using a Hitachi H-8100 electron microscope operated at 200 kV with a LaB6 filament. The samples were dispersed in ethanol and dropped onto a Formvar-coated Cu grid and left to evaporate.
